# Exploring the molecular intersection for hypertension, hyperlipidemia and their comorbid conditions through multi-omics approaches

**DOI:** 10.3389/fcvm.2025.1593688

**Published:** 2025-10-07

**Authors:** Wenjun Li, Dan Zhou, Yanmei Ji, Haitao Tian, Ni Meng, Jisheng Li, Ni Guo, Xianyu He, Mengyao Dao, Xingfang Jin

**Affiliations:** ^1^ Department of Cardiovascular Surgery ICU, Yan'an Affiliated Hospital of Kunming Medical University, Kunming, Yunnan, China; ^2^ Central Laboratory, Yan'an Affiliated Hospital of Kunming Medical University, Kunming, Yunnan, China

**Keywords:** multi-omics, metabolomics, metagenomics, comorbidities, sphingolipids, microbiome, biomarkers

## Abstract

**Background:**

Hypertension and hyperlipidemia are interconnected conditions that heighten cardiovascular risk, yet their intricate multi-scale molecular signatures remain inadequately mapped. This study aimed to conduct an integrated multi-omics investigation to unravel the key pathways and biomarkers underlying hypertension, hyperlipidemia, and both conditions.

**Methods:**

Metabolomic analysis was performed on serum samples and metagenomic analysis on fecal samples collected from individuals with hypertension (*n* = 16), hyperlipidemia (*n* = 19), or both conditions concurrently (*n* = 20). In addition, 20 healthy individuals were recruited as controls.

**Results:**

Metabolomics uncovered altered levels of sphingolipids, phosphatidylcholines, glycylprolines, and nucleic acid metabolites, which may be associated with changes in vascular tone, lipid and protein homeostasis, and thyroid signaling. Metagenomics showed depletion in the abundance of the Fibrobacteres phylum. Altered abundances of *Escherichia coli* and *Bacteroides vulgatus* were also observed, which were correlated with deviations in lipid and carbohydrate metabolism. Sphingomyelin d18:1/16:0 and sphingomyelin d18:1/24:1(15Z) were the key metabolites that were identified as potential diagnostic biomarkers across conditions. Microbial taxa such as *Enterococcus cecorum*, *Lachnospiraceae bacterium*, *Prevotella histicola*, and *Flavobacterium* discriminated these diseases. Pathway analysis revealed glycoxylate, amino acid, purine, and sphingolipid metabolism alterations intersecting hypertension and hyperlipidemia.

**Conclusions:**

This multi-omics landscape of comorbid disease pathways and biomarkers lays the foundation for precision diagnosis and treatment of prevalent cardiovascular conditions.

## 1 Background

Cardiovascular diseases are the leading cause of death worldwide. Hypertension, a prevalent cardiovascular ailment, elevates the cardiac workload and damages vascular walls (1). Hyperlipidemia, on the other hand, fosters atherosclerosis and obstructs blood flow, which therefore escalates the risk of cardiovascular complications (2). Hypertension and hyperlipidemia often coexist and are frequently concomitant in individuals. Both conditions are major risk factors for cardiovascular diseases, and their combined presence can significantly increase the risk of heart disease and stroke (3). Moreover, hypertension and hyperlipidemia are well known to contribute to the development of atherosclerosis, a condition that leads to narrowed and hardened arteries, which reduces blood flow and increases the risk of cardiovascular events (4). Hypertension and hyperlipidemia are common, intertwined conditions that share a significant overlap in underlying risk factors and complications. The comorbidity rate of hypertension and dyslipidemia reached 15.60% in a study of the northwestern Chinese population, which is much higher than that of hypertension and diabetes (4.58%) or diabetes and dyslipidemia (3.57%) (5). The intertwined conditions may collectively hasten the progression of cardiovascular diseases, underscoring the importance of understanding their pathogenesis and identifying effective biomarkers and therapeutic targets for timely intervention.

Innovative research has sought to decode the molecular underpinnings of hypertension, hyperlipidemia, and their concomitant conditions. The serum levels of aldehydes have been found to be associated with hypertension via metabolomics (6). Flavanol, a dietary biomarker, is inversely associated with cardiovascular diseases like hypertension, and its consumption has been shown to effectively reduce blood pressure (7). Disruptions in the tricarboxylic acid cycle and associated metabolic acidosis are hallmark features of early-stage hypertension (8). They may lead to enhanced renal reabsorption of bicarbonate ions (HCO3^−^) and other substances so as to buffer and counteract the excess acidity in the body (9). Key hyperlipidemia markers include triglycerides (TGs), total cholesterol (TC), low-density lipoprotein cholesterol (LDL-C), and high-density lipoprotein cholesterol (HDL-C), which also reflect the metabolic state (10). Carrier proteins such as Apolipoprotein A1 (ApoA1) and Apolipoprotein B (ApoB) are recognized as superior indicators of atherosclerosis, with the ApoB/ApoA1 ratio serving as an effective predictive marker to identify patients with hypercholesterolemia (11, 12).

The onset and progression of hypertension, hyperlipidemia, or both conditions are multifactorial, involving genetic, environmental, and lifestyle factors. Investigating their metabolomics and metagenomics offers deeper insights into disease pathophysiology, and informs clinical strategies for prevention, diagnosis, and management. The multi-scale molecular features of hypertension and hyperlipidemia remain incompletely understood, and current research lacks comprehensive multi-omics integration and sufficient focus on these populations. Here, our study conducts a comprehensive analysis of the blood metabolome and gut microbiome-based metagenome for hypertension, hyperlipidemia, or both conditions. We aimed to examine molecular mechanisms through metabolic biomarkers and microbiota-metabolite interactions. Our research not only explores the molecular mechanisms associated with hypertension, hyperlipidemia, but also provides insights into their comorbidities, offering a more thorough understanding of their underlying biological processes.

## 2 Methods

### 2.1 Participant recruitment and inclusion criteria

Participants aged 40 and above were recruited in this study from Kunming city and Yuxi city, Yunnan Province from September to November 2021. This cohort included a total of 55 patients, which were divided into three groups: 16 patients with hypertension (HT group), 19 with hyperlipidemia (HC group), and 20 with concomitant hypertension and hyperlipidemia (H group). Another 20 age-matched healthy individuals were recruited as controls (CTR group) during the same period from the same area. Participants had not recently taken antibiotics, probiotics, prebiotics, yogurt, or other substances known to significantly affect the gut microbiota. They followed a normal, unrestricted diet and had no history of coronary heart disease, diabetes, cerebrovascular disease, psychiatric disorders, chronic obstructive pulmonary disease (COPD), asthma, malignancy, or dementia. None of the participants were taking lipid-lowering medications or medications for other comorbid conditions. The control group consisted of healthy individuals without any chronic diseases. Patients with abnormal heart, liver, or kidney function and those on long-term medications for blood sugar, or lipid reduction were excluded.

For the HT group, patients with blood pressure ≥140/90 mm Hg (three measurements on different days) were included. Those with secondary hypertension, hyperuricemia, hyperlipidemia, etc. were excluded. For the HC group, patients with total cholesterol levels >5.2 mmol/L and/or triglycerides levels >2.26 mmol/L were included. Patients were recruited in the H group when blood pressure ≥140/90 mm Hg (three measurements on different days), as well as total cholesterol levels >5.2 mmol/L and/or triglycerides levels >2.26 mmol/L.

There were no significant differences in age, sex distribution, smoking, and drinking habits across groups (*p* > 0.05). A written informed consent was acquired from all participants before inclusion. The study was approved by the Medical Ethics Committee of Yan'an Hospital in Kunming, Yunnan Province (2020-096-01).

### 2.2 Sample collection

Fasting blood and fecal samples were collected between 7 and 8 AM following an 8–12 h period without food or drink. Participants were instructed to avoid taking probiotics or antibiotic medication within one month before sample collection. Blood samples were collected on site, centrifuged at 3,500 rpm for 15 min to separate the supernatant, which were used for physiological/biochemical tests and metabolite detection. Fresh fecal samples were collected specifically from the middle to the end of the bowel movement, to reduce environmental contamination and better represent the gut microbial composition. All samples were preserved at −80 °C before analysis.

### 2.3 Physiological and biochemical assays

A total of 48 physiological and biochemical indicators in serum were assessed using a biochemical analyzer. Analysis of variance (ANOVA) was employed to analyze differences among the four groups. Significant indicators (*p* < 0.05) were visualized using box plots with the ggplot2 function (2_3.4.4) in R software (4.3.1).

### 2.4 LC-MS-based (chromatography-mass spectrometry) liquid metabolomics

Metabolites were extracted from 100 μl of each serum sample. First, 400 μl of extraction solution (acetonitrile: methanol = 1:1, with isotopically-labelled internal standard mix) was added and vortexed, followed by sonication in ice-water bath, and incubation at −40 °C for protein precipitation. Samples were then centrifuged at 12,000 rpm for 15 min at 4 °C. The obtained supernatant was collected for analysis, with QC samples prepared by pooling equal volumes of the supernatant of each sample. We used a liquid chromatograph mass spectrometer (UHPLC) system (Vanquish, Thermo Fisher Scientific) equipped with a Waters BEH Amide column, and coupled to a Q Exactive HFX mass spectrometer (Orbitrap MS, Thermo). The mobile phase was composed of (A) 25 mmol/L ammonium acetate and 25 mmol/L ammonia hydroxide in water (pH = 9.75), and (B) acetonitrile. Samples were injected at a volume of 2 μl and maintained at 4 °C in the auto-sampler.

### 2.5 Bioinformatics analysis of metabolomics data

Raw data were converted to mzXML format with ProteoWizard (3.02) and processed with a custom program based on XCMS for peak detection, extraction, alignment, and integration. Metabolites were annotated using an in-house MS2 database (BiotreeDB) with a cutoff value at 0.3. Principal component analysis (PCA) was conducted using the prcomp function (13) in R to elucidate the intrinsic characteristics of the data. *T*-tests was performed to identify differential metabolites (DIMs) via MetaboAnalyst 5.0, and the Benjamini-Hochberg procedure was employed to control the false discovery rate (FDR) (14). Visualization was realized via volcano plots by ggplot2 (2_3.4.4), and heatmaps using pheatmap (1.0.12). Kyoto Encyclopedia of Genes and Genomes (KEGG) pathway enrichment of DIMs was depicted with bubble charts. Random forest algorithm was conducted by MetaboAnalyst 5.0 to classify these metabolites by contribution to classification accuracy and receiver operating characteristic (ROC) curves. Data were auto-scaled (mean-centered and divided by the standard deviation of each variable), followed by automated feature selection and performance evaluation using the random forest algorithm. To assess model performance, Monte Carlo cross-validation (MCCV) with balanced subsampling was applied. In each of the 50 iterations, two-thirds of the data were randomly selected for feature selection and model training, while the remaining one-third was used as an independent test set for performance evaluation. ROC curves and AUC values were calculated solely based on the test data from each iteration. The final performance metrics were averaged across all iterations, with 95% confidence intervals reported. DIMs among multiple groups were analyzed using one-way ANOVA with the aov function in R, and the Benjamini–Hochberg method was applied to control the false discovery rate (FDR).

### 2.6 Metagenomics analysis

Fecal samples were homogenized for microbial release, with genomic DNA extracted for library construction using the NEBNext® Ultra DNA Library Prep Kit (Illumina, USA). Libraries were quantified, pooled, and sequenced on an Illumina PE150 platform. Raw data were quality controlled, assembled, and annotated for species identification against the Nucleotide Collection (NR/NT) database from the National Center for Biotechnology Information (NCBI) (https://www.ncbi.nlm.nih.gov/). PCA and ANOVA were used to analyze gene abundances and identify differential genes and species. The Benjamini–Hochberg procedure was used to control the FDR in ANOVA. The relative abundance of species was charted, and significant species biomarkers were selected using rank-sum tests and LDA (Linear Discriminant Analysis). Finally, gene annotation in KEGG was performed, and correlations between gut microbiome and metabolic pathways were assessed to explore microbial functions. Biomarker selection was facilitated by MetaboAnalyst 5.0 using a random forest algorithm. For metagenomics analysis, one sample was collected from one participant. Alpha diversity at the species level (Chao1, ACE, Shannon, and Simpson indices) and beta diversity (Bray–Curtis distance) were calculated using the vegan package in R. Group differences in alpha diversity were assessed with the Wilcoxon test, while Adonis analysis was applied to evaluate differences in Bray–Curtis distances, reflecting variations in microbial community composition. We used the Virtual Metabolic Human (VMH) database (https://www.vmh.life) to predict the metabolic capabilities of gut microbes, and integrated these predictions with experimentally validated microbe–metabolite–host associations from GutMGene (http://bio-annotation.cn/gutmgene), allowing verification of predicted relationships with experimental evidence. As these resources catalog generic metabolites and species-level or model strains, while our biomarkers included lipid subspecies [e.g., PC(20:1/20:4)] and strain-level taxa, exact curated links were often unavailable. We therefore standardized species to consistent species or genus levels and mapped lipid subspecies to their parent classes to validate the relevant metabolic pathways and reactants.

### 2.7 Statistical power and effect size analysis

For pairwise comparisons, statistical power was estimated using the pwr.t2n.test function in R based on the observed effect sizes and group sample sizes. For overall group comparisons, the pwr.anova.test function in *R* was applied to assess ANOVA-based power. Effect sizes were quantified as Cohen's *d* (using the pooled standard deviation), and 95% confidence intervals were calculated for each metabolite and species-level microbial feature. All analyses were conducted in *R*.

## 3 Results

### 3.1 Variations in physiological and biochemical parameters among groups

**Figure 1:**
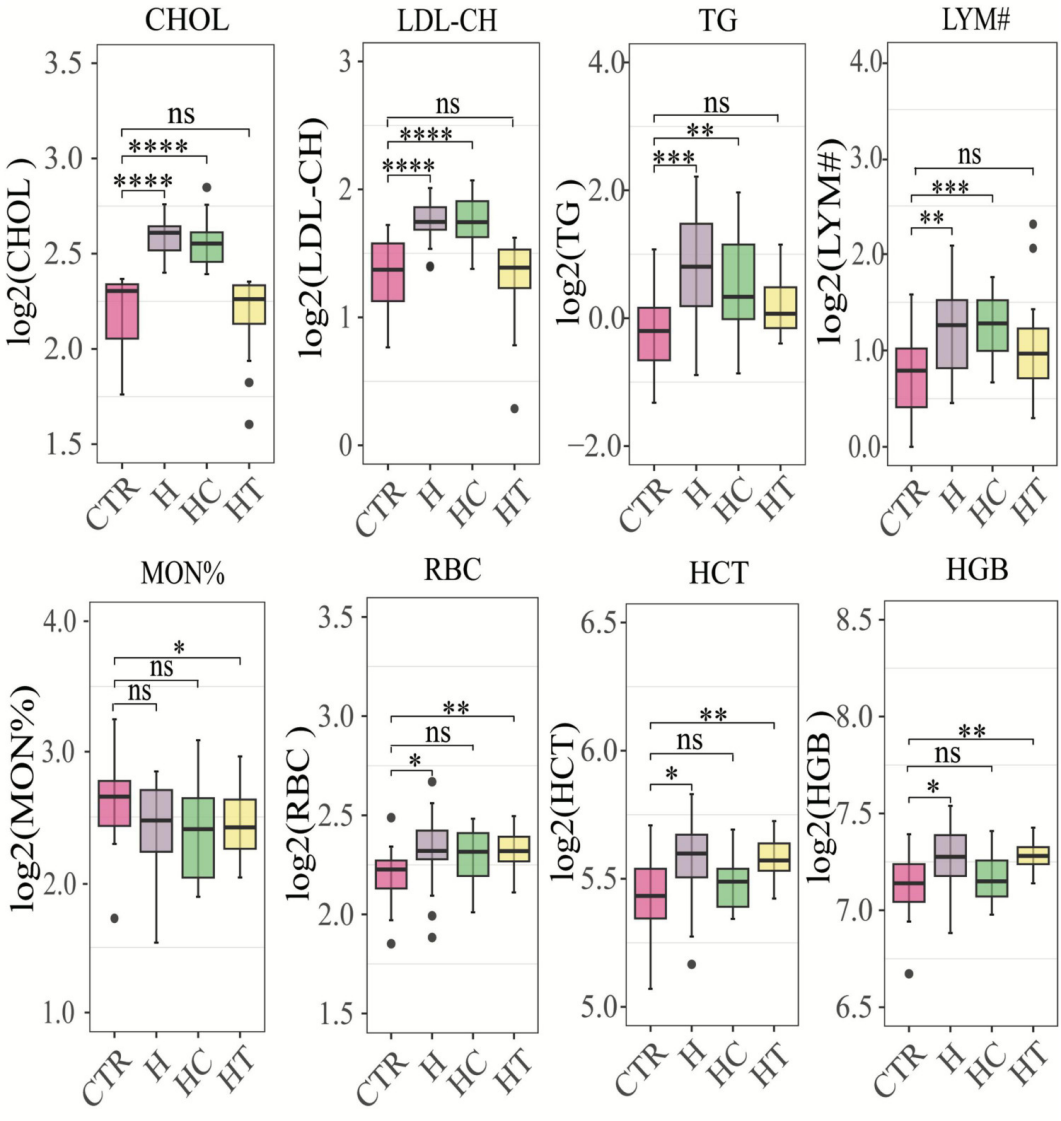
Box plots of significantly different physiological and biochemical parameters. CHOL, cholesterol concentration (mmol/L); LDL-CH, low-density lipoprotein cholesterol concentration (mmol/L); TG, triglyceride concentration (mmol/L); LYM#, represents the number of lymphocytes (10e9/L); MON%, the percentage of monocytes (%); RBC, red blood cell count (10e12/L); HCT, hematocrit (%); HGB, hemoglobin concentration (g/L). *P < 0.05, **P < 0.01, ***P < 0.001, ****P < 0.0001 by the Wilcoxon rank-sum test. CTR: control group; H: concomitant hypertension and hyperlipidemia; HC; hyperlipidemia; HT: hypertension.

In this cross-sectional study, biochemical and hematological parameters were assessed, revealing statistically significant differences in eight distinct biomarkers among groups (Figure 1). Notably, both HC and H groups demonstrated a substantial increase in serum cholesterol (CHOL), low-density lipoprotein cholesterol (LDL-CH), triglycerides (TGs), and lymphocyte count (LYM#), as compared with healthy individuals. Furthermore, the HC group presented with significantly elevated red blood cell count (RBC), hematocrit (HCT), and hemoglobin (HGB) concentration. In the HT group, a notable decrease in monocyte percentage (MON%) was observed, while RBC, HCT, and HGB levels were markedly elevated compared with the control group. These findings underscored the potential interplay between lipid profiles, blood cell parameters, and blood pressure regulation.

### 3.2 Metabolomic profiling reveals differential abundance of metabolites and enriched pathways in disease groups

**Figure 2:**
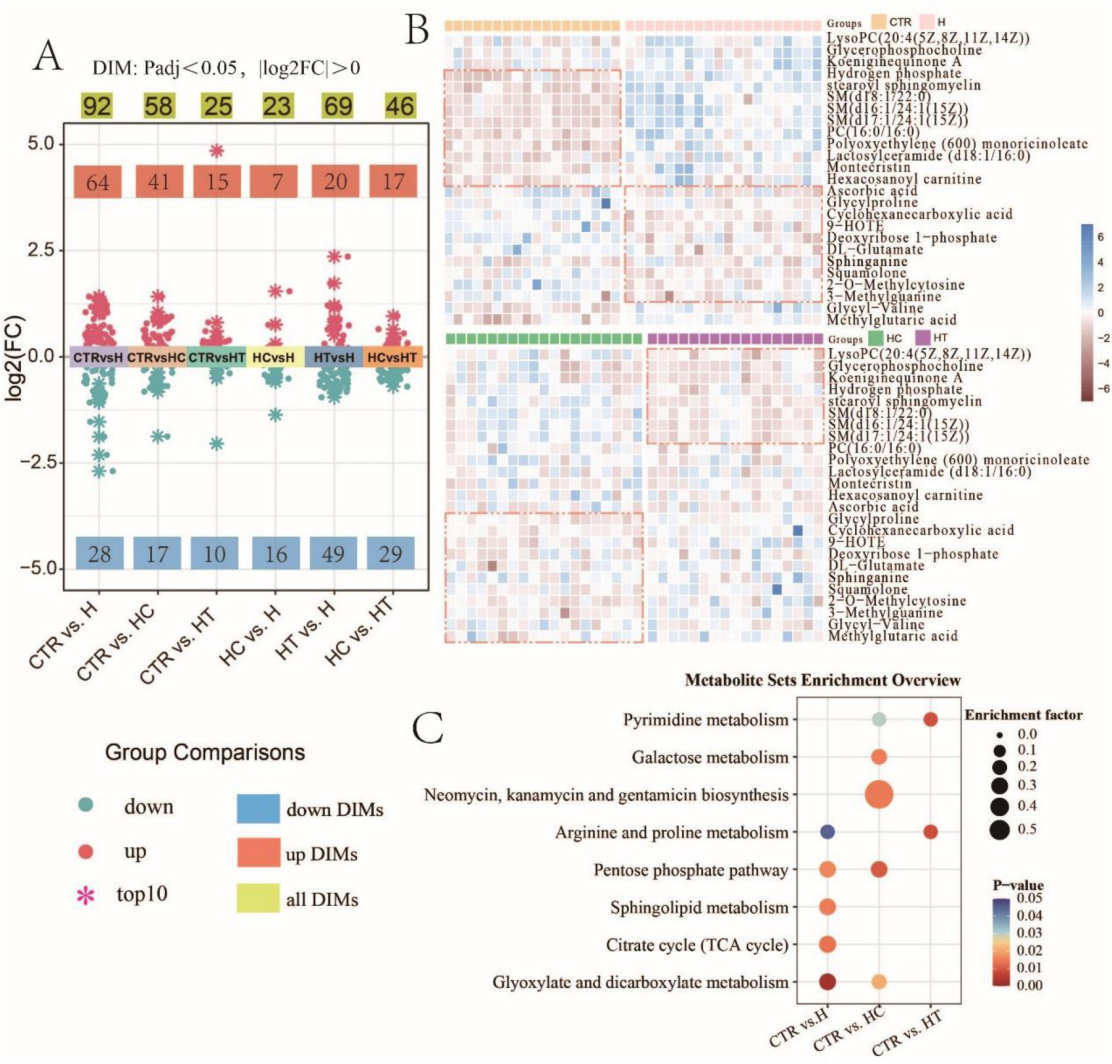
Metabolomic profiling reveals differential abundance of metabolites and enriched pathways in disease groups. **(A)** Distribution of fold changes in group comparisons (Padj < 0.05, |log2FC| > 0). The top 10 largest positive (upregulated) and 10 largest negative (downregulated) log2FC values in each comparison are highlighted with snowflake symbols. **(B)** Heatmap of the top 25 DIMs with the highest relative abundance. DIMs were first identified through pairwise group comparisons. To facilitate visualization, the top 25 DIMs with the highest overall relative abundance across all comparisons were selected for heatmap analysis. **(C)** Bubble chart of significantly enriched metabolic pathways.

To investigate metabolic differences linked to hypertension, hyperlipidemia, and their concurrent conditions, our study embarked on an extensive metabolomic evaluation on the control and disease groups. Differential analysis of metabolites revealed a distinct profile for each patient group compared with the control group. The H group presented with 92 DIMs, with 64 upregulated and 28 downregulated. The HC group exhibited alterations in 58 metabolites, with 41 upregulated and 17 downregulated. The HT group demonstrated changes in 25 metabolites, 15 of which were upregulated and 10 downregulated (Figure 2A). This trend reflected a predominant upregulation of metabolites across all three disease groups, with 11 metabolites shared among the disease–control comparisons (Figure S1A). In contrast, pair-wise comparison among disease groups identified 23 DIMs between HC and H, 69 between HT and H, and 46 between HC and HT (Figure 2A), with only two metabolites overlapping across all three comparisons (Supplementary Figure S1B).

Heatmap analysis of the top 25 most abundant DIMs highlighted metabolites that were upregulated in each disease group. In the H group, elevated levels of ascorbic acid, glycylproline, and cyclohexanecarboxylic acid were observed. The HC group exhibited increased abundance of glycylproline, cyclohexanecarboxylic acid, and 9−HOTE, while the HT group showed notable elevations in LysoPC(20:4(5Z,8Z,11Z,14Z)), glycerophosphocholine, and koeniginequinone A, when compared with the control group (Figure 2B). A one-way ANOVA across the three disease groups identified 11 DIMs, showing an overall tendency for higher levels in the H group (Supplementary Figure S1C). Metabolite set enrichment analysis revealed significant pathway enrichments in each disease cohort. In group H, five pathways were significantly enriched, including glyoxylate and dicarboxylate metabolism, citrate cycle (TCA cycle), sphingolipid metabolism, pentose phosphate pathway, and arginine and proline metabolism. Group HC displayed significant enrichment in several metabolic pathways, including the pentose phosphate pathway, biosynthesis of antibiotics such as neomycin, kanamycin, and gentamicin, galactose metabolism, glyoxylate and dicarboxylate metabolism, and pyrimidine metabolism. In group HT, metabolic alterations were predominantly enriched in two pathways: arginine and proline metabolism and pyrimidine metabolism. Moreover, shared pathway enrichments were identified between groups, such as glyoxylate and dicarboxylate metabolism and the pentose phosphate pathway between group H and group HC; arginine and proline metabolism between H and HT; and pyrimidine metabolism between HC and HT (Figure 2C).

### 3.3 Exploration of serum metabolomic biomarkers

**Figure 3:**
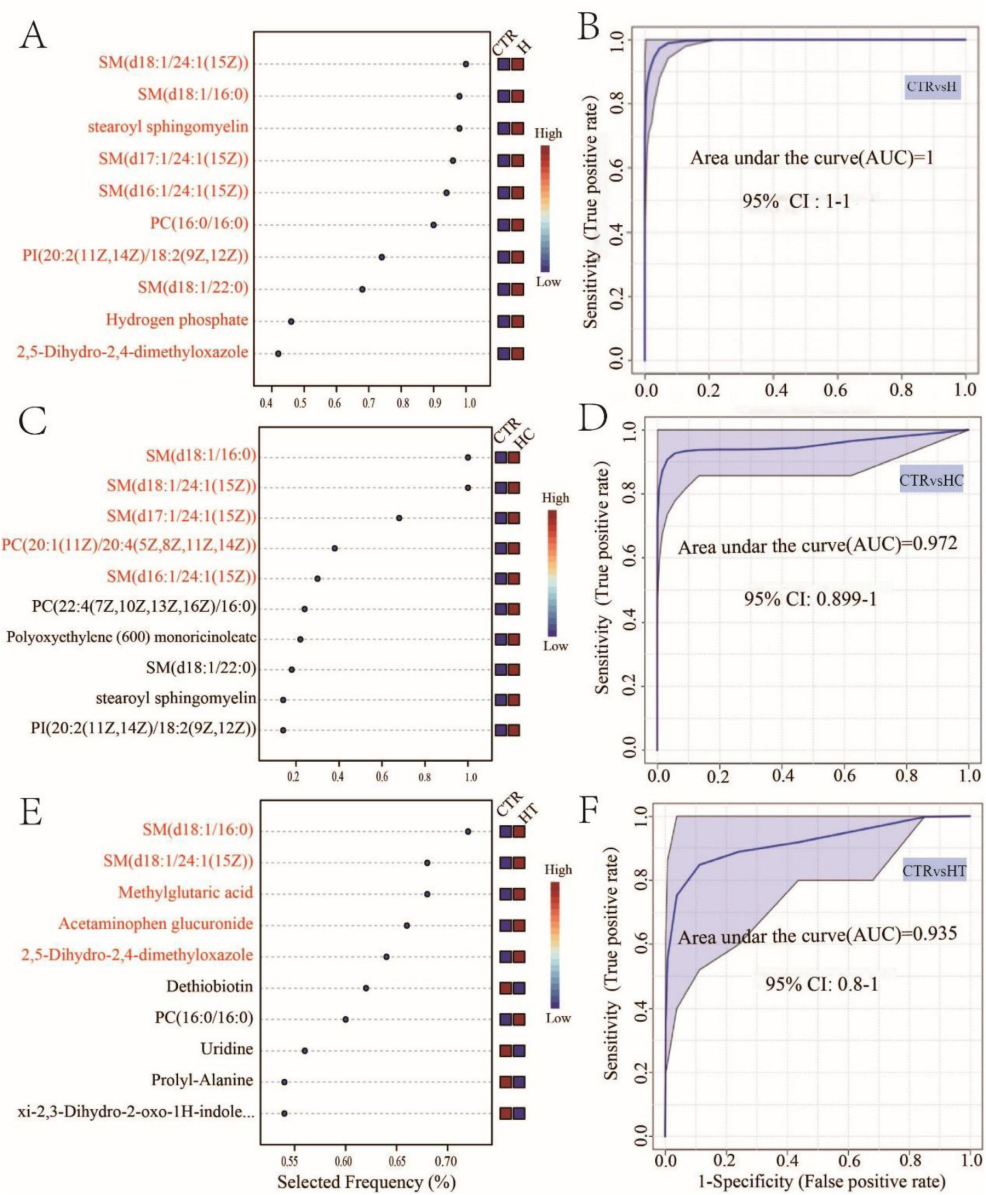
Biomarker analysis of hypertension, hyperlipidemia, and both conditions. Metabolite selection frequency for optimal area under the curve (AUC) between CTR and H **(A)**, CTR and HC **(C)**, and CTR and HT **(E)** Multivariate ROC curves between CTR and H **(B)**, CTR and HC **(D)**, and CTR and HT **(F)**.

To identify potential serum metabolomic biomarkers associated with hypertension, hyperlipidemia, and their comorbid state, a multivariate random forest model was utilized to screen for key discriminatory metabolites. We found that a ten-factor model provided the highest accuracy for distinguishing group H and group CTR, achieving a perfect area under the curve (AUC = 1, Figure 3B). Among the top-ranking metabolites were SM(d18:1/24:1(15Z)), SM(d18:1/16:0), and stearoyl sphingomyelin (Figure 3A). In the comparison between HC and CTR, a five-factor model yielded the highest AUC (AUC = 0.972, Figure 3D), with metabolites such as SM(d18:1/16:0), SM(d18:1/24:1(15Z)), and SM(d17:1/24:1(15Z)) were predominantly selected (Figure 3C). Additionally, when HT and CTR were compared, a five-factor model with an AUC of 0.935 was identified (Figure 3F). Metabolites like SM(d18:1/16:0), SM(d18:1/24:1(15Z)), and methylglutaric acid emerged as key discriminatory metabolites (Figure 3E). These findings underscored the potential application of serum metabolomic biomarkers in the diagnosis of hypertension, hyperlipidemia, and both conditions. Notably, SM(d18:1/16:0) and SM(d18:1/24:1(15Z)) were consistently identified across all three disease groups. Their recurrent selection underscores their potential as shared metabolic signatures associated with cardiovascular and lipid-related pathophysiology.

### 3.4 A metagenomic insight into gut microbiome variances among groups

To delve deeper into gut microbiome alterations associated with hypertension, hyperlipidemia, and their comorbidity, we performed metagenomic sequencing of fecal samples from each participant. PCA revealed a similarity in gene abundance patterns across groups (Supplementary Figure S2A), while the correlation heatmap further confirmed the repeatability of the biological samples (Supplementary Figure S2B). Non-redundant genes (Supplementary Figure S2C) and shared genes (Supplementary Figure S2D) between groups were also detected. We compared the alpha diversity among groups and found that for Shannon and Simpson, the control group exhibited the highest diversity, while the H group showed the lowest (Supplementary Figure S3A). Furthermore, beta diversity analysis revealed significant differences in community composition among the groups (adonis: *R*^2^ = 0.163, *P* < 0.05) (Supplementary Figure S3B).

**Figure 4:**
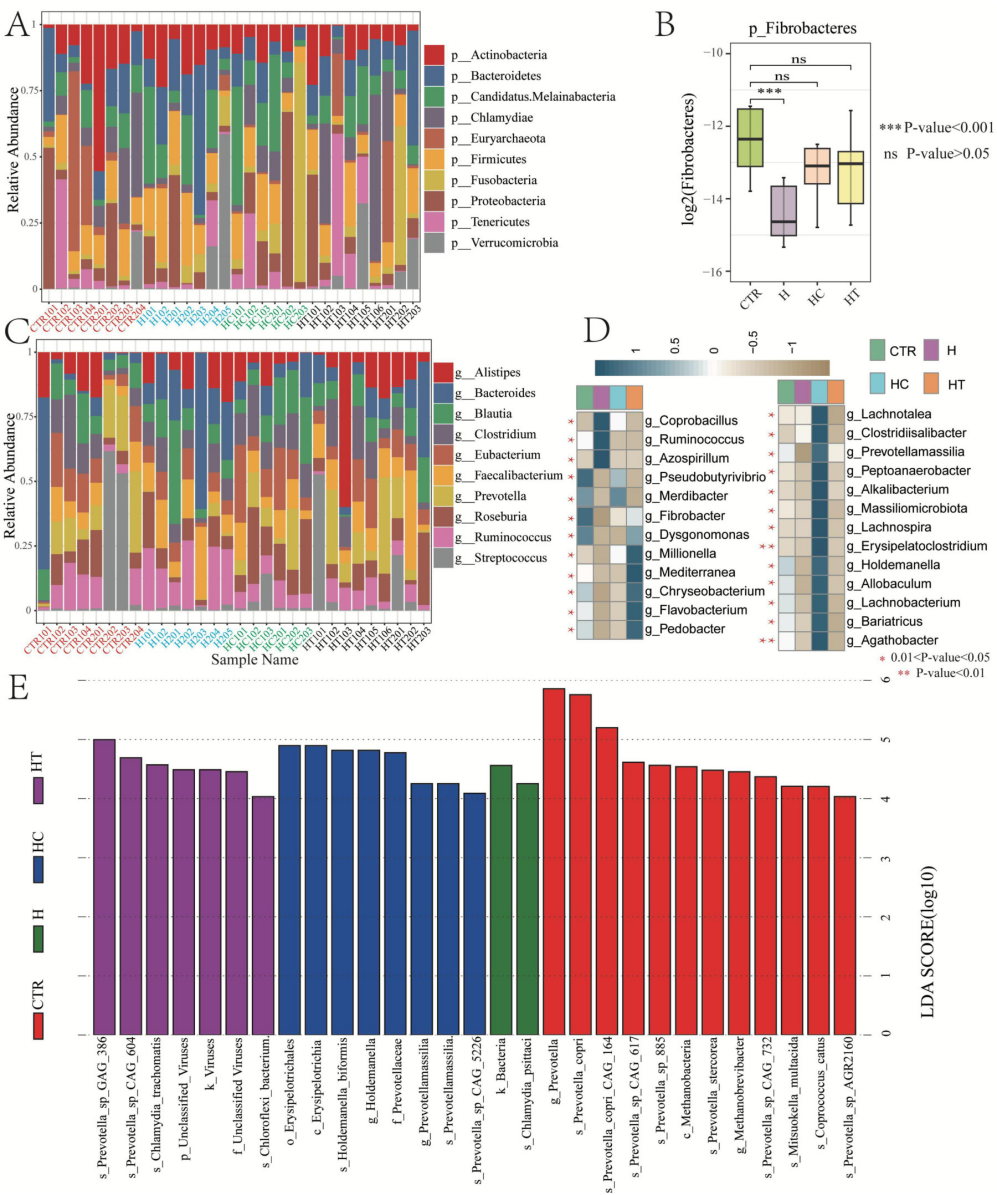
Gut microbiome variances among groups. **(A)** Bar chart showing relative abundances of top 10 microbial phyla. **(B)** Box plot showing the relative abundance variances of Fibrobacteres among groups. **(C)** Bar chart showing relative abundances of top 10 microbial genera. **(D)** Heatmap showing the relative abundance of differential microbial genera across groups. E: Distribution of linear discriminant analysis (LDA) scores for differential microbial taxa (LDA score >4).

At the phylum level, the microbial composition and structure were similar across the four groups, with some degree of intra-group differences. Actinobacteria and Bacteroidetes showed distributions in the majority of samples across all groups (Figure 4A). Only Fibrobacteres showed an inter-group difference, with a significantly lower relative abundance in the H group compared with the CTR group. However, no differences were observed between the HC and HT groups (Figure 4B).

At the genus level, both intra-group and inter-group differences were observed. Notably, the H group harbored a higher relative abundance of *Prevotella* compared with other groups. The HC group had a higher abundance of *Roseburia* but lower abundance of *Ruminococcus* compared with the CTR and H groups. The HT group was higher in the abundance of *Faecalibacterium*, but lower in *Ruminococcus* (Figure 4C). Overall, 25 genera exhibited significant differences in relative abundance across groups. Specifically, *Pseudobutyrivibrio* and *Fibrobacter* were significantly enriched in the control group. *Coprobacillus*, *Ruminococcus*, and *Azospirillum* were more abundant in the H group, while *Pseudobutyrivibrio*, *Merdibacter*, and *Lachnotalea* were enriched in the HC group. The HT group showed significantly higher abundances of genera such as *Millionella*, *Mediterranea*, *Chryseobacterium*, *Flavobacterium*, and *Pedobacter*, compared with other groups (Figure 4D).

LEfSe analysis identified distinct differential taxa across all taxonomic levels in each disease group. In the H group, two differential taxa were detected, namely Bacteria and *Chlamydia psittaci*. The HC group demonstrated eight differential taxa, including Erysipelotrichales, Erysipelotrichia, and *Holdemanella biformis*. In contrast, the HT group had seven, such as *Prevotella* sp. GAG_386, *Prevotella* sp. CAG_604*,* and *Chlamydia trachomatis* (Figure 4E).

### 3.5 Discrepancies in metabolic functions across groups

**Figure 5:**
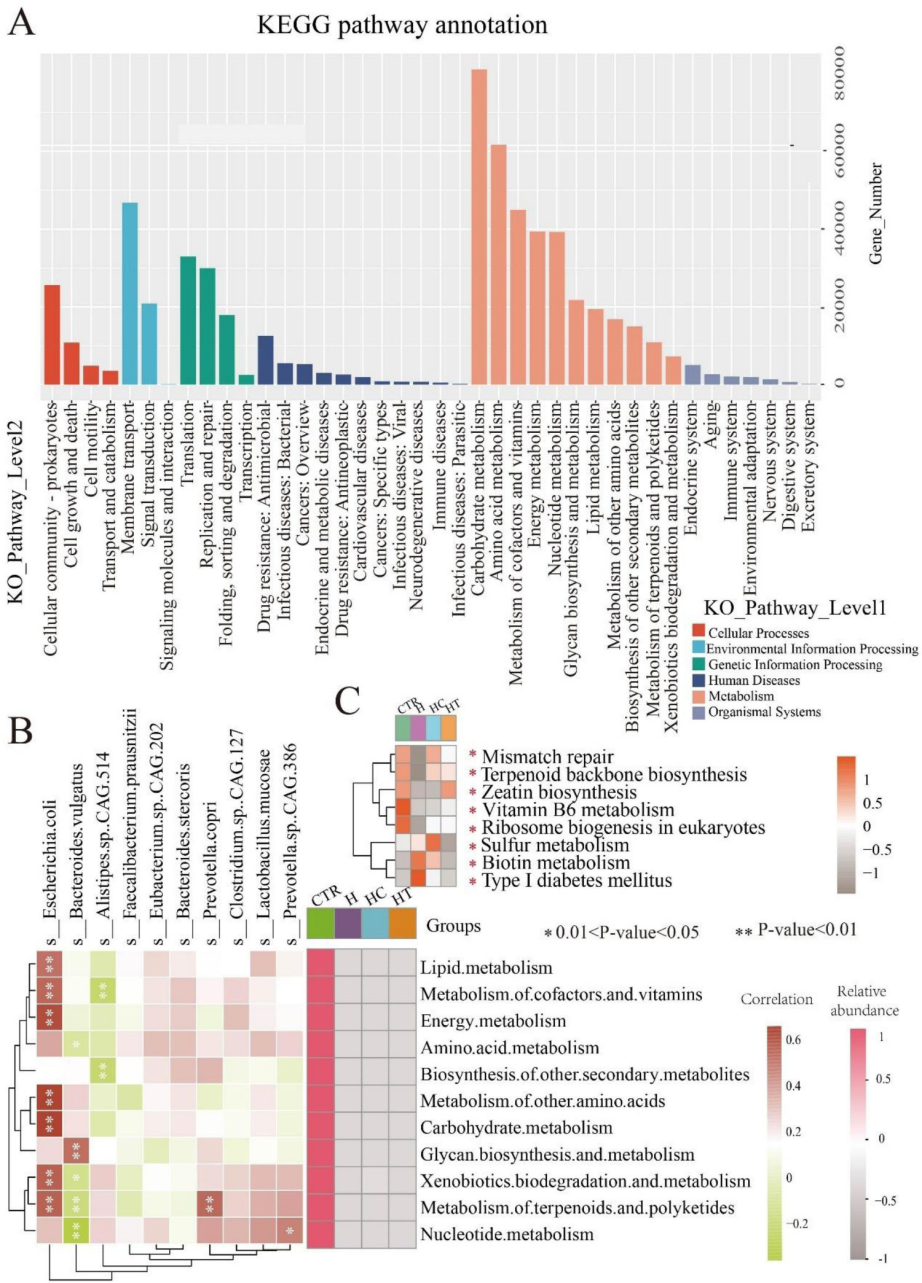
Metabolic function distinctions among groups. **(A)** KEGG microbial gene pathway annotation. **(B)** Heatmap showing the correlation between the top 10 microbial species and secondary pathways within the metabolism category, alongside the relative abundance of these secondary pathways. Left heatmap: correlation between microbial species and pathways, with color blocks representing correlation strength (red: positive; green: negative). *P* < 0.05, *P* < 0.01. Right heatmap: relative abundance of secondary pathways, with color intensity representing average relative abundance (Pink colors indicate higher abundance). **(C)** Heatmap of significantly enriched pathways at Level 3. Color intensity represents average relative abundance, with darker colors indicating higher relative abundance. *P < 0.05.

Functional annotation and pathway analysis were performed on the metagenomes to investigate differences in metabolic functions among the microbial communities. KEGG-based annotation revealed gene assignments across six primary level 1 categories, namely: cellular processes, environmental information processing, genetic information processing, human diseases, metabolism, and organismal systems. At the secondary level, these categories encompassed 4, 3, 4, 11, 11, and 7 pathways, respectively. Notably, the metabolism category exhibited the greatest number of annotated secondary pathways and genes (Figure 5A). Subsequent correlation analysis between secondary metabolism pathways and microbial species revealed several significant associations. For instance, *E. coli* exhibited strong correlations with lipid metabolism and six additional pathways. *Bacteroides vulgatus* was related to glycan biosynthesis and metabolism, along with four other pathways. *Alistipes* sp. CAG.514 showed correlations with metabolism of cofactors and vitamins, as well as biosynthesis of other secondary metabolites. *Prevotella copri* was significantly correlated with metabolism of terpenoids and polyketides, while *Prevotella* sp. CAG.386 with nucleotide metabolism. Notably, these pathways exhibited lower relative abundance in the three disease groups than in the control group (Figure 5B), suggesting that their correlated microorganisms might influence the metabolic pathway functions associated with these diseases. At level 3, eight pathways exhibited significant differences among groups. In the H group, mismatch repair, terpenoid backbone biosynthesis, zeatin biosynthesis, vitamin B6 metabolism, and ribosome biogenesis in eukaryotes were suppressed, while sulfur metabolism, biotin metabolism, and Type I diabetes mellitus pathways were activated. The HC group showed similar suppression of zeatin biosynthesis, vitamin B6 metabolism, and ribosome biogenesis in eukaryotes, alongside activation of sulfur and biotin metabolism pathways. In the HT group, mismatch repair, vitamin B6 metabolism, and ribosome biogenesis in eukaryotes pathways were suppressed (Figure 5C).

### 3.6 Identification of gut microbiome biomarkers using multivariate random forests

**Figure 6:**
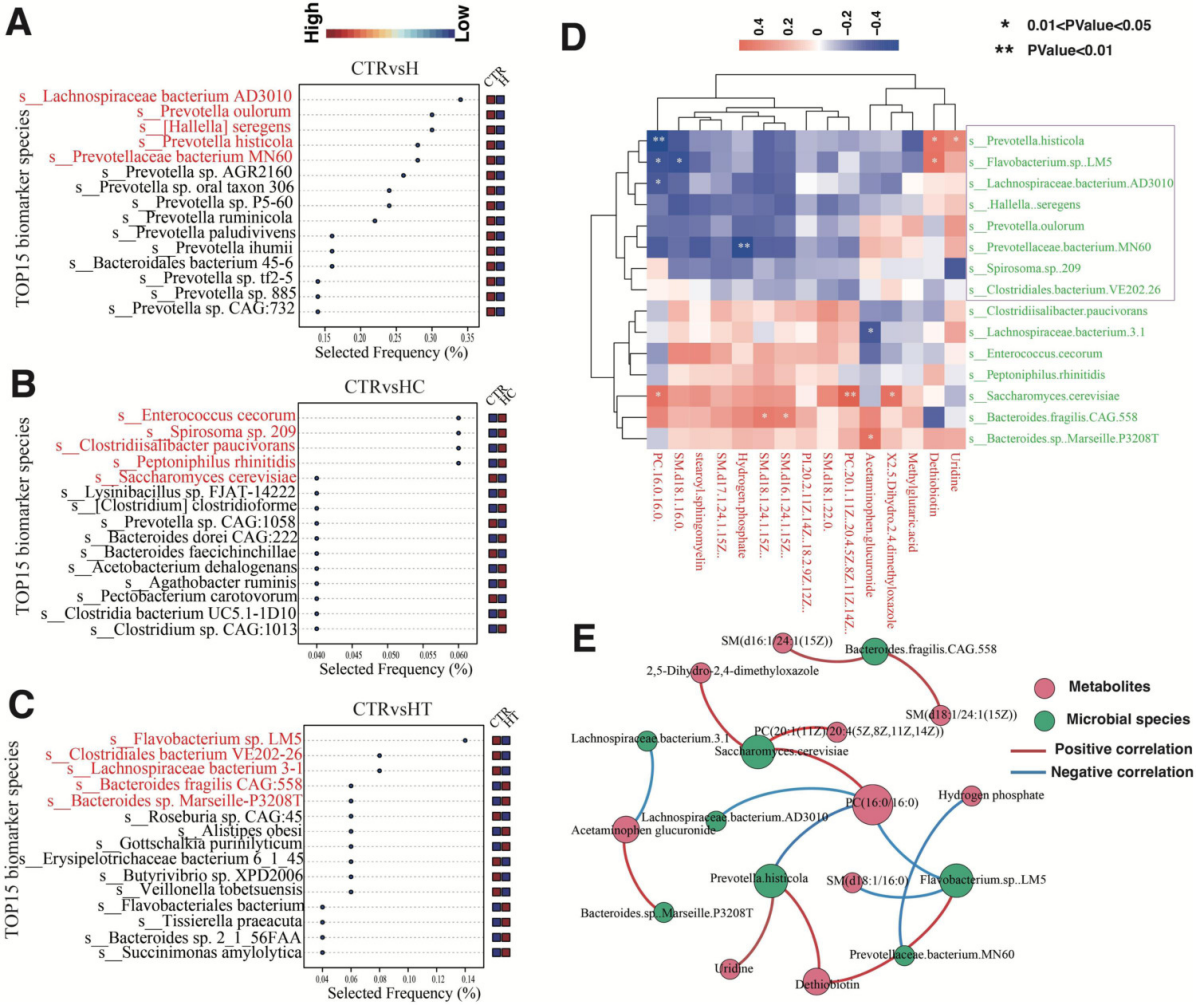
Detection of gut microbiome biomarkers. **(A–C)** Microbial selection frequency for optimal area under the curve (AUC) between CTR and H **(A)**, CTR and HC **(B)**, and CTR and HT **(C,D)** Heatmap of correlation analysis between key differential microbial species and differential metabolites. The color blocks denote the strength of correlation, with red indicating a positive correlation and blue signifying a negative correlation. **(E)** Correlation network analysis between key differential microbial species and differential metabolites. Nodes represent microbial species (green) and metabolites (red), while edges indicate significant correlations (*P* < 0.05). Red edges denote positive correlations, and blue edges represent negative correlations. The size of each node corresponds to the strength (degree) of its associations, with larger nodes indicating higher connectivity.

To further identify fecal microbiome biomarkers for hypertension, hyperlipidemia, and their comorbidity, we employed a multivariate random forest model at the species level to pinpoint key microbial species. A five-factor model demonstrated the highest classification accuracy in distinguishing the H group from the CTR group (AUC = 1). Compared with the control group, *Lachnospiraceae bacterium* AD3010, *Prevotella oulorum*, *Hallella seregens*, *Prevotella histicola*, and *P. bacterium* MN60 were frequently selected in the H group, suggesting their potential as efficient microbial markers to differentiate healthy individuals from those with concurrent hypertension and hyperlipidemia (Figure 6A). In the HC group, species such as *E. cecorum*, *Spirosoma* sp. 209, *Clostridiisalibacter paucivorans*, *Peptoniphilus rhinitidis*, and *Saccharomyces cerevisiae* exhibited high selection frequencies, indicating their potential roles in identifying and predicting hyperlipidemia (Figure 6B). For the HT group, *Flavobacterium* sp. LM5, *Clostridiales bacterium* VE202-26, *Lachnospiraceae bacterium* 3-1, *Bacteroides fragilis* CAG:558, and *Bacteroides* sp. Marseille-P3208T were frequently selected, which may serve as microbial markers distinguishing hypertensive patients from healthy individuals (Figure 6C).

We then performed correlation analysis between the identified key microbial biomarkers and previously detected metabolite biomarkers. The results revealed notable positive correlations between *S. cerevisiae* and PC (20:1(11Z)/20:4(5Z,8Z,11Z,14Z)), *B. sp. Marseille-P3208T* and acetaminophen glucuronide, and *F sp. LM5* and dethiobiotin. In contrast, negative correlations were observed between *P. histicola* and PC(16:0/16:0), *P. bacterium MN60* and hydrogen phosphate, as well as *L. bacterium 3-1* and acetaminophen glucuronide (Figures 6D–E, Supplementary Table S1). These findings suggest potential associations between these microbial species and specific metabolite levels. Significant microbe–metabolite pairs were also cross-checked against VMH (Supplementary Table S2) and GutMGene (Supplementary Table S3). Collectively, these findings suggest that the gut microbiota may exhibit distinct enrichment patterns among groups, and these associations may provide preliminary evidence of biologically plausible connections to disease phenotypes.

To assess the influence of sample size and group effects on statistical power, we conducted a one-way ANOVA power analysis based on the average group size (power ≈ 0.95, assuming an effect size *f* = 0.5), as well as pairwise comparisons assuming a mean difference of 0.8 standard deviations (power = 0.64–0.68). These results suggest that the current sample size provides sufficient power to detect large effect sizes, but may be underpowered for medium or small effects. Detailed results of the power analysis are provided in Supplementary Table S4.

## 4 Discussion

Hyperlipidemia principally refers to elevated serum levels of total cholesterol, triglycerides, and low-density lipoprotein (LDL), or reduced high-density lipoprotein (HDL) levels. This study found that compared with patients with hyperlipidemia alone, those with concomitant hypertension and hyperlipidemia exhibited not only the typical hyperlipidemic profile of high cholesterol and triglycerides, but also slightly elevated LDL levels, suggesting that hypertension may exacerbate lipid abnormalities. Moreover, changes in lymphocyte and monocyte levels may reflect alterations in immune regulatory mechanisms, while variations in red blood cell count and hemoglobin concentration correlated with changes in hemorheology (15). Significant shifts in these indicators in concomitant hyperlipidemia and hypertension suggested that this particular medical condition may engage multiple metabolic and immune regulatory pathways, influencing the physiological state of the blood.

Clinically, hypertension and hyperlipidemia manifest metabolic imbalance, and our study discovered that the differential metabolites predominantly related to lipid, protein, and nucleic acid metabolism. Individuals characterized by lower serum levels of lysophosphatidylcholine (PC), a biomarker of hypertension, face a 3.8-fold increased risk of early vascular aging compared with those with higher levels (16). Differential metabolites identified in this study, such as phosphatidylethanolamines (PEs), phosphatidylinositols (PIs), phosphatidylcholines (PCs), and sphingomyelins (SMs), are common in sphingolipid metabolism. For instance, PCs were found to be associated with endothelial function, potentially through regulation of endothelial-derived nitric oxide, a vasodilator involved in maintaining vascular tone (17). Additionally, PCs are involved in triglyceride synthesis and metabolism, which may influence cholesterol transport and overall lipid balance, which in turn influences blood triglyceride levels (18, 19). PIs, a universal signaling molecule, were associated with cell activity by directly interacting with membrane proteins such as ion channels and G protein-coupled receptors (GPCRs), or by recruiting cytoplasmic proteins to the membrane. These processes likely involve thyroid hormone signaling (20). SMs, a key component of the cell membrane, have been reported to be involved in the synthesis and release of thyroid hormones (21). Previous studies suggest that impairments in the release and signaling of thyroid hormones could lead to hypothyroidism, which may contribute to the development of hypertension and hyperlipidemia (22, 23). Protein metabolism is primarily reflected in the synthesis of amino acids such as glycine, proline, and glutamate, which are involved in protein and lipid synthesis. These amino acids influence the transport of cholesterol and triglycerides, and are intimately connected with the excretion and reabsorption of sodium (24). Certain proteins, especially peptides, may regulate vascular tension, affecting vascular relaxation and contraction. For example, neuropeptides like angiotensin and catecholamines play non-negligible roles in hypertension development (25, 26). Metabolites involved in nucleic acid metabolism, such as 2−O−Methylcytosine and 3−Methylguanine, primarily modify bases and DNA. Nucleic acid is associated with lipid metabolisms, especially in the purine and pyrimidine pathways, where their metabolites can influence lipid biosynthesis and degradation (27). The differential metabolites between disease groups and healthy individuals were significantly enriched in pathways related to amino acid metabolism, purine metabolism, and glycolipid metabolism. These findings suggest that these metabolites may influence disease progression through lipid, protein, and nucleic acid metabolic pathways, especially during the pre-symptomatic phase.

In exploring the gut microbiome composition of patients with hypertension, hyperlipidemia, and both conditions, we discovered a notable decrease in the abundance of a particular microbial phylum—Fibrobacteres—in the H group. An imbalance in gut microbiota may be associated with immune and metabolic diseases (28). We hypothesized that hyperlipidemia may cause dysregulation in the immune system and metabolism, particularly lipid metabolism, thus leading to a lower abundance of Fibrobacteres (29). At the genus level, common genera such as *Clostridiisalibacter*, *Allobaculum*, and *Bariatricus* can produce beneficial short-chain fatty acids (SCFAs), such as propionate, butyrate, and acetate. These SCFAs play a crucial role in the breakdown of complex polysaccharides, thereby facilitating their intestinal absorption (30). Gut microbiota dysbiosis can lead to the secretion of large amounts of amyloid proteins and lipopolysaccharides, which have been implicated in conditions like obesity, type 2 diabetes, and hyperglycemia, all of which are associated with lipid dysregulation (28, 31). Most genera showed differential abundance in the HC group, leading us to conjecture that gut microbiota profoundly influence hyperlipidemic diseases. This is particularly true for genera involved in polysaccharide breakdown or lipopolysaccharide secretion, as their overabundance may be a risk factor for the development of hyperlipidemia.

Metabolic functions were the most affected by alterations in the microbiome in patients from the disease groups. Specifically, *E. coli*, *B. vulgatus*, and an unidentified species of *Alistipes* were significantly related to multiple pathways, including lipid metabolism, carbohydrate metabolism, and energy metabolism. *E. coli*, one of the most common bacterial species in the human gut, plays in a vital role in the decomposition and absorption of food. It aids the host in nutrient acquisition and vitamins K/B complex synthesis. In addition, *E. coli* also modulates immune responses and maintains intestinal immune balance by interacting with host immune system (32). *B. vulgatus* engages in the fermentation of carbohydrates, utilization of nitrogenous substances, and biotransformation of bile acids and other sterols. It maintains homeostasis and prevents bacterial and viral infections through immune regulation (33). Further correlation analysis revealed that lipid metabolites, particularly PCs and SMs, were more susceptible to the influence of gut microbiota. Notably, Gram-negative genera such as *Prevotella*, *Flavobacterium*, and *Prevotellaceae* exhibited negative correlations with PCs and SMs, whereas Gram-positive *Clostridiisalibacter*, *Peptoniphilus*, and *Enterococcus* showed positive correlations. These differences may be attributed to the distinct cell wall and membrane structures between Gram-negative and Gram-positive bacteria, with PCs and SMs being major components of their cell membranes. In particular, lipopolysaccharides in the outer membrane of Gram-negative bacteria might interact with membrane components like phosphatidylcholine, potentially influencing cellular signaling and immune responses (34, 35). While this observation may represent a novel mechanistic link between microbial composition and host lipid metabolism, further studies are necessary to validate this hypothesis. Moreover, we identified notable positive correlations between specific microbes and metabolites, including *S. cerevisiae* with PC (20:1(11Z)/20:4(5Z,8Z,11Z,14Z)), *B.* sp. Marseille-P3208T with acetaminophen glucuronide, and *F*. sp. LM5 with dethiobiotin. It has been reported that *S. cerevisiae* can synthesize fatty acids, a key component in lipid synthesis (36, 37). Acetaminophen glucuronide is known to participate in liver metabolism, particularly in glucuronidation and sulfation pathways (38). This suggests the possibility that *B.* sp. Marseille-P3208T is linked to host metabolic processes. In summary, our findings highlight significant correlations between gut microbiota and metabolites relevant to hypertension, hyperlipidemia, and their coexistence, suggesting potential but non-causal links between microbial composition and host metabolic pathways.

Our study provides a preliminary analysis of the molecular mechanisms underlying hypertension, hyperlipidemia, and their comorbidities through multi-omics approaches. However, several limitations should be noted. First, the relatively small sample size with uneven group distribution may reduce the statistical power and limit the generalizability of our findings. Future studies with larger, independent cohorts are warranted to validate the identified features and confirm the robustness and predictive value of our models. Second, factors such as recent dietary intake, circadian rhythms, and physical activity were not uniformly controlled, which may have introduced variability in the metabolomic and microbiome profiles. Future studies should adopt more standardized sampling protocols to minimize these sources of biological variation. Third, this study employs a cross-sectional design, which limits the ability to infer causal relationships of diseases. While differences in metabolite levels, microbial community composition, and other factors have been identified between the disease and control groups, it remains unclear whether these differences are causes or consequences of the diseases. Longitudinal studies are needed to elucidate the directionality and potential mechanistic relevance of these associations. Finally, this study lacks functional validation of key findings. While *Flavobacterium* sp. LM5 was identified as a hypertension biomarker, *in vitro* experiments are required to confirm its role in hypertensive patients.

## 5 Conclusions

In conclusion, our multi-omics investigation revealed notable molecular signatures across the blood metabolome and gut microbiome that are associated with hypertension, hyperlipidemia, and their comorbid conditions. Key DIMs were linked to disruptions in sphingolipid, amino acid, and nucleotide metabolism, which may be related to changes in vascular tone, lipid homeostasis, and thyroid signaling. Reduced abundances of beneficial gut microbes like Fibrobacteres and enriched species including *E. coli* and *B. vulgatus* were correlated with deviations in lipid and carbohydrate metabolism. These integrated omics analyses provide a systems-level view of the molecular pathways driving these medical conditions, offering potential diagnostic biomarkers and illuminating targets for therapeutic manipulation. It must be emphasized that the identified metabolites and microbial taxa are candidate biomarkers and need functional validation before any clinical application can be considered. Further multi-omics examination encompassing broader patient cohorts can serve to validate and refine the mechanistic models towards enabling personalized therapeutic interventions. Overall, our work lays the foundation for unraveling the intricate disease networks underlying these prevalent cardiovascular risks, offering insights into risk stratification, diagnosis, and treatment outcomes.

## Data Availability

The original contributions presented in the study are publicly available. The raw metagenomic data can be found in the NCBI Sequence Read Archive (SRA) under the BioProject accession number PRJNA1277202. The metabolomics data can be found in the MetaboLights repository under the accession number MTBLS13039.
