# Supplementary material for: Exploring the molecular intersection for hypertension, hyperlipidemia and their comorbid conditions through multi-omics approaches
Source: Front Cardiovasc Med. 2025 Oct 7;12:1593688. doi: 10.3389/fcvm.2025.1593688 (PMC12537794; doi:10.3389/fcvm.2025.1593688)
Supplement: Supplementary file 5 [file Datasheet1.pdf]

Table legends

Table S1. Correlation coefficients between key microbial species and previously identified key metabolites.

Table S2. Carbon source utilization profiles of significant microbe–metabolite pairs.

Table S3. Significant microbe–metabolite pairs and their host-associated effects recorded in GutMGene.

Table S4. Statistical power and effect size analysis for pairwise and group comparisons.

Supplementary figures

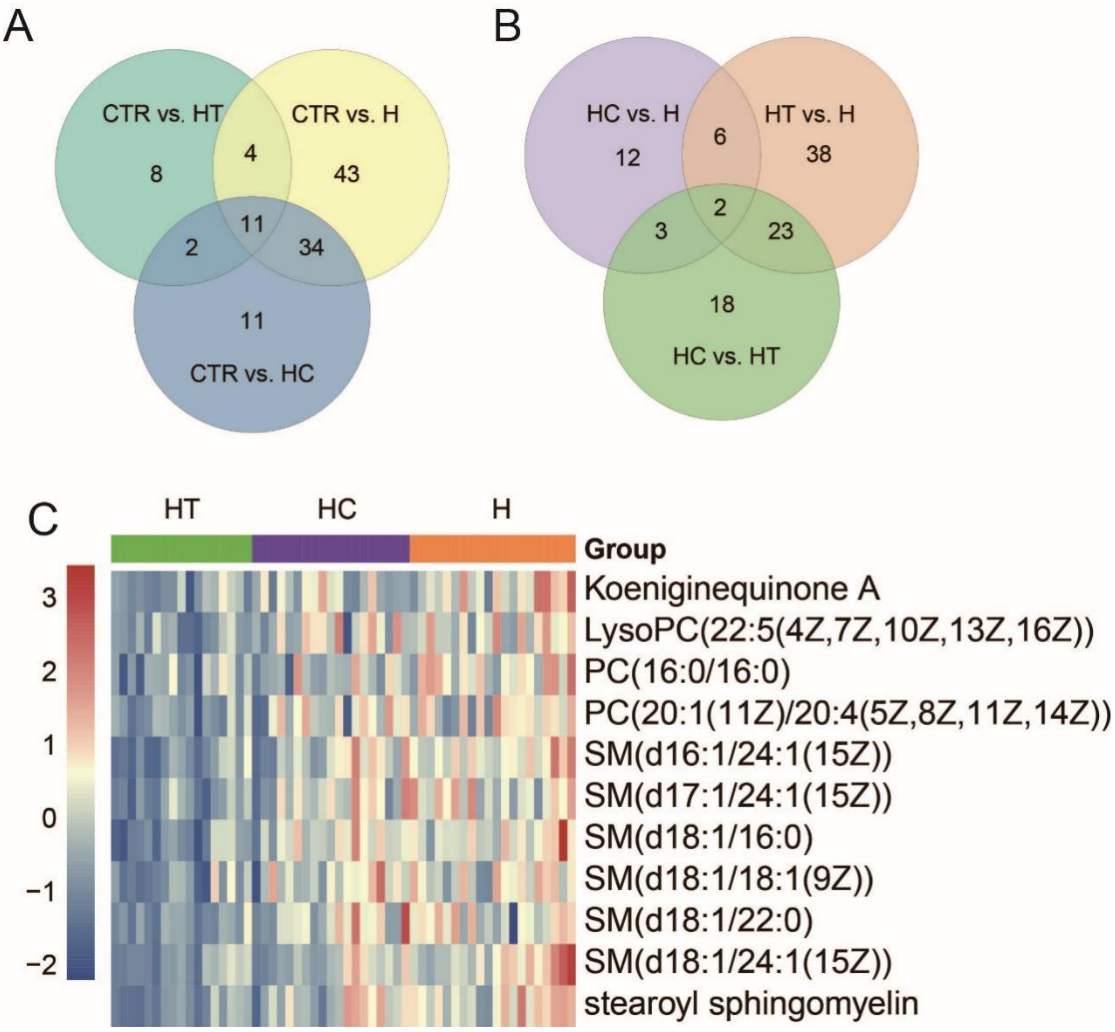

Figure S1. Analysis of differential metabolites (DIMs) between and among groups. A: Venn diagrams depicting the number of DIMs between the control group and each

disease group. B: Venn diagram showing the number of DIMs shared between the disease groups. C: Heatmap illustrating the accumulation patterns of DIMs among the three disease groups, as identified by one-way ANOVA.

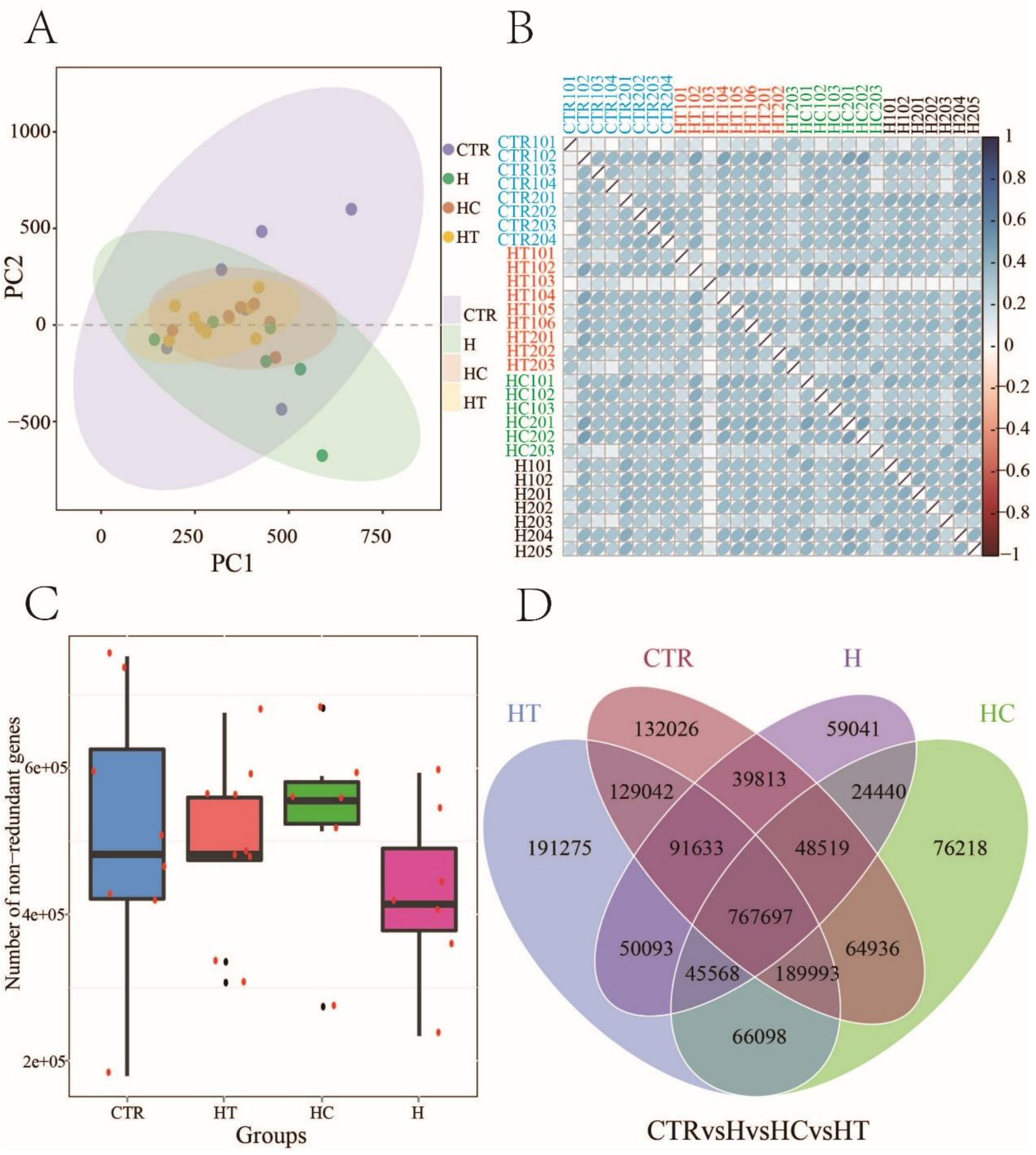

Figure S2. Overview of metagenomic data of each group. A: Principal component analysis (PCA) of the metagenomes. B: Heatmap depicting sample correlations, with color intensity representing Spearman correlation coefficients: blue indicates positive correlation, and red indicates negative correlation. C: Non-redundant gene analysis. D: Number of shared genes between groups.

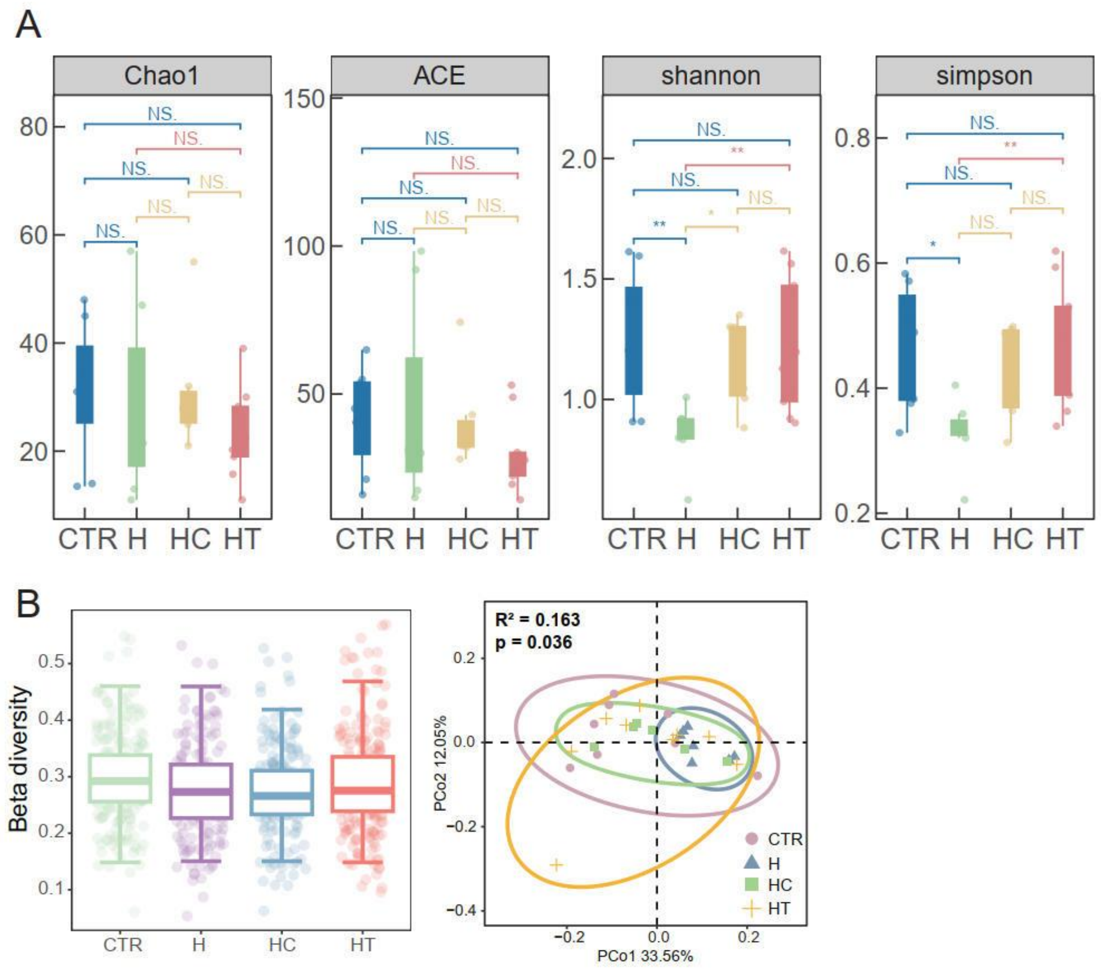

Figure S3. Diversity analysis of metagenomic data among groups. A: Alpha diversity comparisons among groups, including Chao1, ACE, Shannon, and Simpson indices. B: Beta diversity comparisons among groups, visualized by principal coordinates analysis (PCoA) based on Bray–Curtis dissimilarity, accompanied by a box plot. Statistical differences were assessed using PERMANOVA (adonis), with $R^2 = 0.163$ and $P < 0.05$.
